# Membrane Condensation and Curvature Induced by SARS-CoV-2 Envelope Protein

**DOI:** 10.1021/acs.langmuir.3c03079

**Published:** 2024-01-23

**Authors:** Christian Wölk, Chen Shen, Gerd Hause, Wahyu Surya, Jaume Torres, Richard D. Harvey, Gianluca Bello

**Affiliations:** † Pharmaceutical Technology, Medical Faculty, University Leipzig, Eilenburger Straße 15a, 04317 Leipzig, Germany; ‡ Deutsches Elektronen-Synchrotron DESY, Notkestr. 85, 22607 Hamburg, Germany; § Biocenter, Martin-Luther University Halle-Wittenberg, Weinbergweg 22, 06120 Halle (Saale), Germany; ∥ School of Biological Sciences, Nanyang Technological University, 50 Nanyang Avenue, Singapore 639798, Singapore; ⊥ Division of Pharmaceutical Chemistry, Department of Pharmaceutical Sciences, University of Vienna, Josef-Holaubek-Platz 2, UZA 2, Vienna 1090, Austria

## Abstract

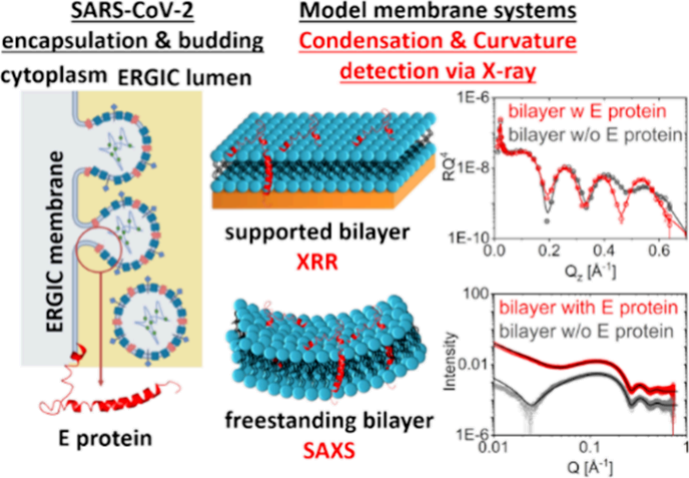

The envelope (E) protein of SARS-CoV-2 participates in virion encapsulation and budding at the membrane of the endoplasmic reticulum Golgi intermediate compartment (ERGIC). The positively curved membrane topology required to fit an 80 nm viral particle is energetically unfavorable; therefore, viral proteins must facilitate ERGIC membrane curvature alteration. To study the possible role of the E protein in this mechanism, we examined the structural modification of the host lipid membrane by the SARS-CoV-2 E protein using synchrotron-based X-ray methods. Our reflectometry results on solid-supported planar bilayers show that E protein markedly condenses the surrounding lipid bilayer. For vesicles, this condensation effect differs between the two leaflets such that the membrane becomes asymmetric and increases its curvature. The formation of such a curved and condensed membrane is consistent with the requirements to stably encapsulate a viral core and supports a role for E protein in budding during SARS-CoV-2 virion assembly.

## Introduction

Of the four structural proteins encoded by SARS-CoV-2, the nucleocapsid (N), spike (S), matrix (M), and envelope (E), it is the latter which has proved the most elusive with respect to defining its precise role in the viral infection cycle.^1^ Although the biology of the E protein in SARS-CoV-2 has not yet been examined in detail, it is most probably very similar to those of other coronaviruses, in particular to that of SARS-CoV-1 because their sequences are almost identical (SARS-CoV-1:76 amino acids versus SARS-CoV-2:75 aa),^2,3^ with the exception of one deletion and two conservative substitutions. The 76 amino acid long E protein of SARS-CoV-1 localizes in the endoplasmic reticulum–Golgi intermediate compartment (ERGIC) of infected cells^4^ where virus morphogenesis and budding occur. It has a cytoplasmically oriented C-terminus and permeabilizes membranes to ions^5−9^ by forming pentameric oligomers^10,11^ mediated by a single α-helical transmembrane domain (TMD).^2^ The structure of this viroporin channel in synthetic ERGIC membranes has been published recently at high resolution.^3^ Microscopy studies have shown that the presence of E protein in the ERGIC membrane is required for virion release from the host cell.^1,12^ Additionally, E protein evidently participates in coronavirus virion budding into the lumen of the ERGIC,^12−14^ which suggests that it plays a role in inducing membrane curvature.^1^ In coronaviruses, the minimum requirement for production of virus-like particles (VLP) with similar morphology to infectious virions is the co-expression of M and E by the host cell.^14^ Coronavirus M protein alone does not appear to affect host cell internal membrane structure,^15^ whereas E-deletion mutants lead to the accumulation of morphologically defective attenuated virions inside host cells.^12^ This indicates a putative pivotal role for coronavirus E proteins in the promotion of ERGIC membrane curvature, a phenomenon which has been observed in molecular dynamics simulations, but nevertheless remained unproven experimentally.^16,17^

Devising experiments to understand E protein/lipid interactions requires starting with simple model systems that represent the intracellular membranes of the secretory pathway, with a focus on physiologically relevant alkyl chain lengths that mimic the lipid bilayer thickness of biomembranes, e.g., C16 (palmitoyl) and C18-Δ9 (oleoyl). In contrast to previous *in vitro* studies that have focused on the channel structure of E protein in model ERGIC membranes,^2,3^ we have chosen to examine structural changes in its lipid host matrix induced by E protein, as these may shed light on the role of E protein in virion budding. The model membranes we used have compositions which mimic some key characteristics of the ERGIC lipid environment. The use of various phosphatidylcholine (PC)/phosphatidylserine (PS) mixtures satisfied the need for a model that was slightly anionic,^3,18^ fully miscible,^19^ and which alone would not exhibit any curvature bias.^20^ Bilayer models composed of both mixed saturated/monounsaturated palmitoyloleoyl (PO) acyl chains and fully saturated dipalmitoyl (DP) chains provide a simple model for testing E protein against loosely packed ER and ERGIC membranes (POPC/POPS) versus more tightly packed Golgi or plasmalemma bilayers (DPPC/DPPS).^20^

To assess the influence of SARS-CoV-2 E protein on various membrane structural features, we used X-ray reflectometry and small-angle scattering, as they provide angstrom-level resolution of lipid bilayer structures.^21,22^ We combined these techniques with cryo-transmission electron microscopy to examine whether alterations in bilayer structure attributable to coronavirus E protein can affect lipid morphologies on the microscopic scale. Our results reveal differential asymmetry-inducing effects of E protein on different model systems, which suggest that both lipid charge density and packing behavior play important roles in tempering membrane responses to E protein interactions.

## Materials and Methods

### SARS-CoV-2 Envelope Protein Purification and Characterization

A SARS-CoV-2 E protein construct with N-terminal 6-His and MBP tag was derived from its SARS-CoV-1 counterpart^6^ by introducing T55S, V56F, E69R, and G70del mutations by site-directed mutagenesis. A plasmid carrying this construct was transformed into *E. coli* BL21-CodonPlus (DE3)-RIPL (Agilent). The cells were cultivated by fed-batch method with K12 media^23^ in a 1 L fermenter (Winpact) at 37 °C. A 40% dissolved oxygen saturation was maintained by stirring, aeration, and O_2_ supplementation. Protein expression was induced with 0.5 mM IPTG at the same time as feeding was started, and cultivation continued at 18 °C overnight. The culture was harvested by centrifugation at 7500*g*. E protein was purified from the cell pellet by Ni-NTA chromatography and reverse-phase-HPLC as described previously.^6^ The dry E protein (*M*_w_ = 8541.12 g/mol) powder was then dissolved in ethanol (denatured, ≥99.8%, Carl Roth GmbH) at a concentration of 1 mg/mL.

### Preparation of Lipid Stock Solutions

Stock solutions of 1,2-dipalmitoyl-*sn*-glycero-3-phosphatidylcholine (DPPC), 1,2-dipalmitoyl-*sn*-glycero-3-phosphatidyl-l-serine (sodium salt, DPPS), 1-palmitoyl-2-oleoyl-PC (POPC), and POPS (sodium salt) in chloroform/methanol mixture (7:3 vol/vol, both HPLC grade from Merck KGaA) were prepared by dissolving the powdered lipids as purchased (Avanti Polar Lipid) to 1.5 mM concentration. The PC and PS stocks were mixed at a 90:10 volume ratio to obtain the 1.5 mM stock solution for the binary lipid mixtures (PC:PS, 90:10 mol/mol). The PC:PS 95:5 (mol/mol) mixture was prepared by combining the PC stock solution and 90:10 stock at a 1:1 volume ratio.

### Liposome Dispersion Preparation

The buffer solution in Milli-Q water containing 10 mM Tris and 0.05 mM EDTA was preadjusted to pH 7.4 with hydrochloric acid (solutes from Merck KGaA). The different PC and PS lipid stock solutions (organic solvent as described above) with and without E protein (dissolved in ethanol) were mixed in a round-bottom flask to achieve the correct PC/PS/protein molar ratio and dried under rotary evaporation (45 °C, 25 mbar, 45 min). They were then rehydrated with the buffer to 22 mg/mL for 2 h, at room temperature for PO-liposomes and at 45 °C for DP-liposomes and the liposomes with protein, followed by vortexing. The samples with protein were additionally vortexed and sonicated for 5 min (PO/E) and 15 min (DP/E) at 45 °C.

### X-ray Reflectometry on Solid Supported Bilayers

Structures of supported lipid bilayer (SLB) samples were measured by X-ray reflectometry at beamline P23 at PETRA III (DESY, Germany), using an unfocused 18 keV beam of 0.25 mm × 0.40 mm (*h* × *v*) size. Specular reflection and the background at *Q*_*xy*_ = 0.06 Å^–1^ on both sides were measured by a Lambda 750k GaAs detector (X-Spectrum, Germany) mounted at 0.87 m distance from the sample after an 8.0 mm × 0.5 mm (*h* × *v*) postsample slit at 0.2 mm from the sample.

The (100) silicon wafer (9.0 mm × 15.0 mm, 0.6 mm thick, Si-Mat, Kaufering, Germany) supports for SLB deposition were cleaned with an RCA-1 protocol^24^ and kept in Milli-Q water for no more than 6 days. Prior to the deposition, the wafer was mounted into the SDU-Odense membrane chamber (AG. Klösgen, University of Southern Denmark, Denmark). The chamber was filled with 1 mL of the buffer and equilibrated at the deposition temperature (22 °C for POPC and 53 °C for DPPC). Diluted liposome dispersion (2 mg/mL) was sonicated for 30 min at the same temperature prior to deposition. An injection of 500 μL of the dispersion was made into the buffer bulk just above the wafer, using a preheated syringe, followed by 30 min incubation at the deposition temperature. The final concentration and the volume in the chamber were 0.67 and 1.5 mL, respectively. Thereafter, the chamber was flushed with 5 mL of buffer. The chamber was then thermostatically adjusted to the measurement temperature for about 10 min prior to the X-ray measurement. The real-time temperature of the aqueous phase was monitored over the preparation and measurement by an integrated Pt100 sensor. E protein adsorption experiments were performed on a pure lipid sample that had been measured at different positions. About 0.5 mL of buffer was taken out from the chamber after the film deposition to be premixed with the 0.05 mL of E protein stock solution. The mixture was reinjected back into the chamber just above the wafer. During this process, the wafer was always immersed in the buffer. Immediately after the injection, the sample was measured every 40 min for 5 h.

**Figure 1:**
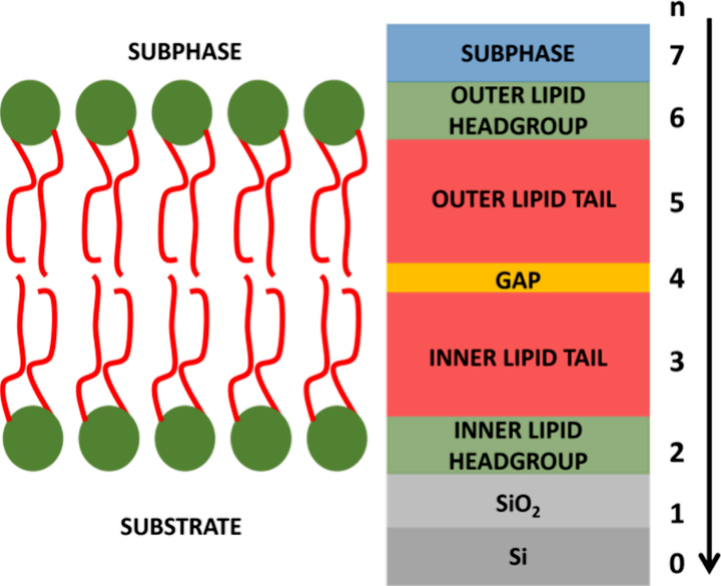
Slab compartment model for a supported lipid bilayer. Six slab compartments (no. 1–6) are used between the substrate (0) and the subphase (7).

The X-ray data were calibrated by the critical angle, corrected by the background and footprint effect, and normalized to the total reflection, to yield the reflectivity curves. The structure of the supported bilayer was modeled by the slab-compartment model (Figure 1) for fitting the measured reflectivity data.^25^ The system between the silicon substrate and the water-based subphase was differentiated into six slabs with different X-ray scattering length density (SLD, ρ_b_). Except for the outer headgroup region (6), each slab (1–5) starts with an interface where the volume filling fraction of the previous compartment decreases as an error function from 100% to 0%, while the volume filling fraction of this slab increases to 100% accordingly. The volume filling is then 100% until the end of the slab, where the filling decreases as an error function to 0%, with an increased volume filling of the material of the next slab. The outer headgroup region also started with an error function increase at its interface from the outer tail region until the volume fitting reaches 100% at the three times of the error function width. Thereafter, the volume filling drops as a half-Gaussian peak to zero, while the subphase volume filling increases to 100%. The SLD profile ρ_b_(*z*) of the system is therefore the volume weighted sum of the SLD of all the slabs. The theoretical reflectivity from the model is calculated as^25^
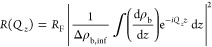
namely, the Fourier transformation on the variation of the scattering length density (SLD, ρ_b_) on the depth *z*, normalized by the Fresnel reflectivity *R*_F_ and the difference Δρ_b,inf_ of the SLD between the silicon substrate and the aqueous subphase. The fitting is done on log *RQ*^4^ up to 0.65 Å^–1^ and is accepted when the summed deviation is minimized below 0.4, in order to avoid a physically unreasonable local minimum. The thickness of the hydrophobic core *d*_core_ was then quantified as the distance between the two headgroup–tail interfaces, while the total thickness of the bilayer was defined as the distance *d*_HH_ between the centroids of the two headgroup compartments. The area *Â* per lipid in the SLB was further obtained from the SLD of the hydrophobic core region as *Â* = *r*_e_·2*N*_e,t_/∫_*z*_6,5__^*z*_3,2_^ρ_b_(*z*) d*z*, where 2*N*_e,t_ is the total number of electrons in the tail region of two lipids and *r*_e_ = 2.8 × 10^–15^ m is the classical electron radius. The integration of the scattering length density runs from position *z*_6,5_ of the higher headgroup–tail interface to position *z*_3,2_ of the deeper headgroup–tail interface. A custom written Matlab script was used for all data analysis.

### Small-Angle X-ray Scattering on Liposome Dispersions

The membrane structure in the liposomes was measured with small-angle X-ray scattering (SAXS) experiments by the EMBL BioSAXS beamline P12 at PETRA III (DESY, Germany)^26^ using the 22 mg/mL liposome stock dispersions as well as the buffer blank at 22.3 °C. The measurements were done at 10.0 keV with a beam size of 0.20 mm × 0.12 mm (*h* × *v*), using a Pilatus 6M detector (Dectris, Switzerland) at a 3 m distance. The data were initially calibrated to the absolute scale by using pure water at 22.3 °C, with buffer background subtracted and normalized by the lipid concentration, using the automated data processing pipeline DATOP with DATABSOLUTE module.^27^

The SAXS data were analyzed by a recently developed volumetric approach to obtain the area *Â* per lipid in each leaflet, the bilayer SLD profile at absolute scale (Å^–2^), and the volume of the tail region per lipid; while the details of this routine can be found elsewhere,^28^ a brief description is given here. The SLD contrast Δρ_b_^′^(*z*) of the bilayer against water on an arbitrary scale is first obtained by fitting the SAXS data with a five-slab-compartment model of the bilayer, combined with the modified Caillé theory in the presence of Bragg peaks in the data.^29^ The five-slab-compartment model is slightly different from the one used for the supported bilayer in that the two headgroup/bulk interfaces are both half-Gaussians. This model also includes asymmetry with respect to the distributions of the two leaflets. The internal contrast variations between the headgroup and the core region is uniquely associated with the scaling factor to the absolute SLD value once the volume *V*_CGP_ of the carbonyl–glycerophosphate (CGP) group excluding the hydration. This value was measured by grazing incidence X-ray off-specular scattering on monolayers of the same lipid composition with the same buffer at the high-resolution diffraction beamline P08 at PETRA III to be 242 Å^3^ for PC and 248 Å^3^ for PC/PS membrane (90:10).^28^ The area per lipid is obtained first as 
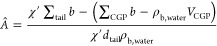
where 
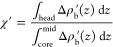
 is the ratio between the integrated SLD contrast of the head and that of one tail leaflet. *d*_tail_ is the thickness of one tail leaflet. ∑_tail_*b* and ∑_CGP_*b* are the total scattering lengths of the tail region and the CGP region of one lipid, respectively. The water SLD is ρ_b,water_ = 9.42 × 10^–6^ Å^–2^. Then identical scaling factors for the SLD value should be obtained as 
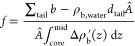
 from both leaflets. The absolute SLD is then calculated as ρ_b_(*z*) = *f*Δρ_b_^′^(*z*) + ρ_b,water_. Again Matlab was used for full data analysis.

### Circular Dichroism Spectroscopy on Liposome Dispersions

Circular dichroism (CD) experiments for the determination of the secondary structure of the E protein were performed on a Chirascan instrument (Applied Photophysics, Surrey, UK) at the SPC facility of the EMBL laboratories (Hamburg, Germany).

The samples from the SAXS experiments were diluted in buffer to 1:10 of the original concentration and measured three times in a 1 mm path length quartz cell within the wavelength range 185–260 nm. The spectrum of the buffer was subtracted. To account for the light scattering induced by the lipid vesicles, spectra from the vesicles without peptide were subtracted, and the data were factorized in relation to the absorbance at 215 nm, within the range of 210–220 nm at which the protein absorption is lowest and the lipid absorption is highest.^30,31^ The data were converted into mean residue ellipticity ([θ]MR) and smoothed using a Savitzky–Golay filter at 5 points of window. The percentage of secondary structure (Figure S3) was calculated using the Dichroweb analysis web server.^32^

### Cryo-Transmission Electron Microscopy on Liposome Dispersions

Vitrified specimens for cryo-TEM were prepared using a blotting procedure, performed in a chamber with controlled temperature and humidity using an EM GP grid plunger (Leica, Wetzlar, Germany). The sample dispersion (6 μL) was placed onto an EM grid coated with a holey carbon film (Cflat, Protochips Inc., Raleigh, NC). Excess solution was then removed by blotting (12 s) with filter paper to leave a thin film of the dispersion spanning the holes of the carbon film on the EM grid. Vitrification of the thin film was achieved by rapid plunging of the grid into liquid ethane held just above its freezing point. The vitrified specimen was kept below 108 K during storage, transferred to a microscope, and investigated. Specimens were examined with a Libra 120 Plus transmission electron microscope (Carl Zeiss Microscopy GmbH, Oberkochen, Germany), operating at 120 kV. The microscope was equipped with a Gatan 626 cryo-transfer system. Images were acquired using a BM-2 k-120 dual-speed on-axis SSCCD camera (TRS, Moorenweis, Germany).

### Differential Scanning Calorimetry (DSC)

The DSC measurements of 1–3 mg/mL liposome dispersions (with and without incorporated E protein) were performed on a MicroCal Peaq-DSC (Malvern Panalytical, Northampton, MA). The temperature range was scanned between 10 and 60 °C; the heating rate was 60 K/h, and each heating and cooling scan was repeated three times to check for reproducibility. Medium feedback mode was used for the data recording. The reference cell was filled with pure buffer, and the buffer–buffer baseline was subtracted from the thermograms of the vesicle samples. The DSC scans were analyzed using MicroCal Origin 8.0 software.

## Results and Discussion

**Figure 2:**
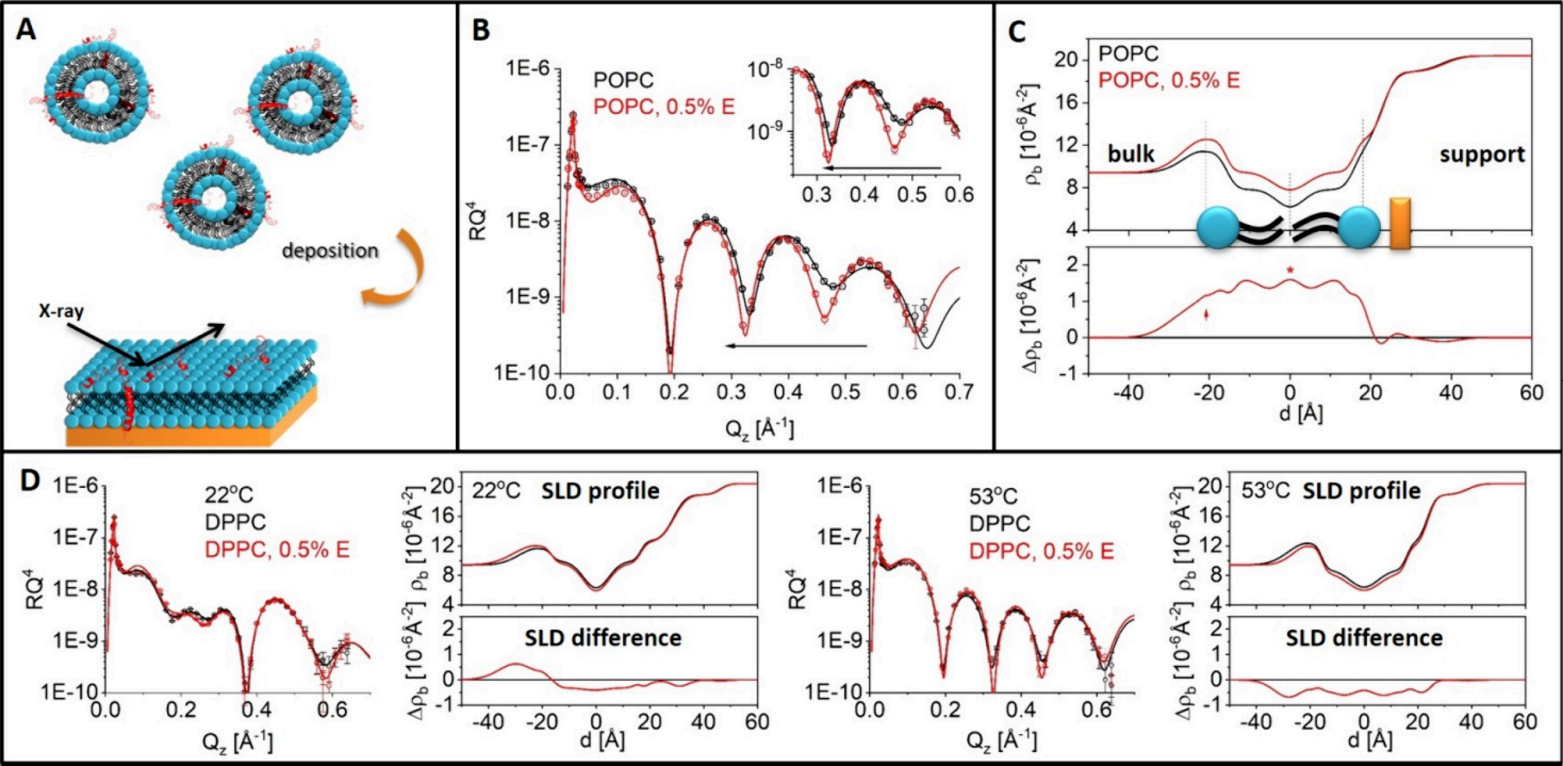
XRR and SLD profiles of the co-deposited SLB with E protein. (A) Schematic illustration of the SLB co-deposition experiment with lipid vesicles incorporating E protein in the lipid bilayer. (B) Reflectivity curve (symbols) and fitted curve (solid line) of a POPC SLB in the presence and absence of 0.5 mol % E protein. The arrow indicates the significant shift of the Kiessig fringe. (C) SLD profiles of SLBs resulting from the fitting in C (upper panel) and the SLD difference of both curves (lower panel). The model of the lipid bilayer is shown to guide the eye. (D) Reflectivity curve (symbols) and fitted curve (solid line) of a DPPC SLB in the presence and absence of 0.5 mol % E protein at 25 and 53 °C. The SLD profiles of the SLBs resulting from the fitting and the SLD difference of both curves are also shown.

**Figure 3:**
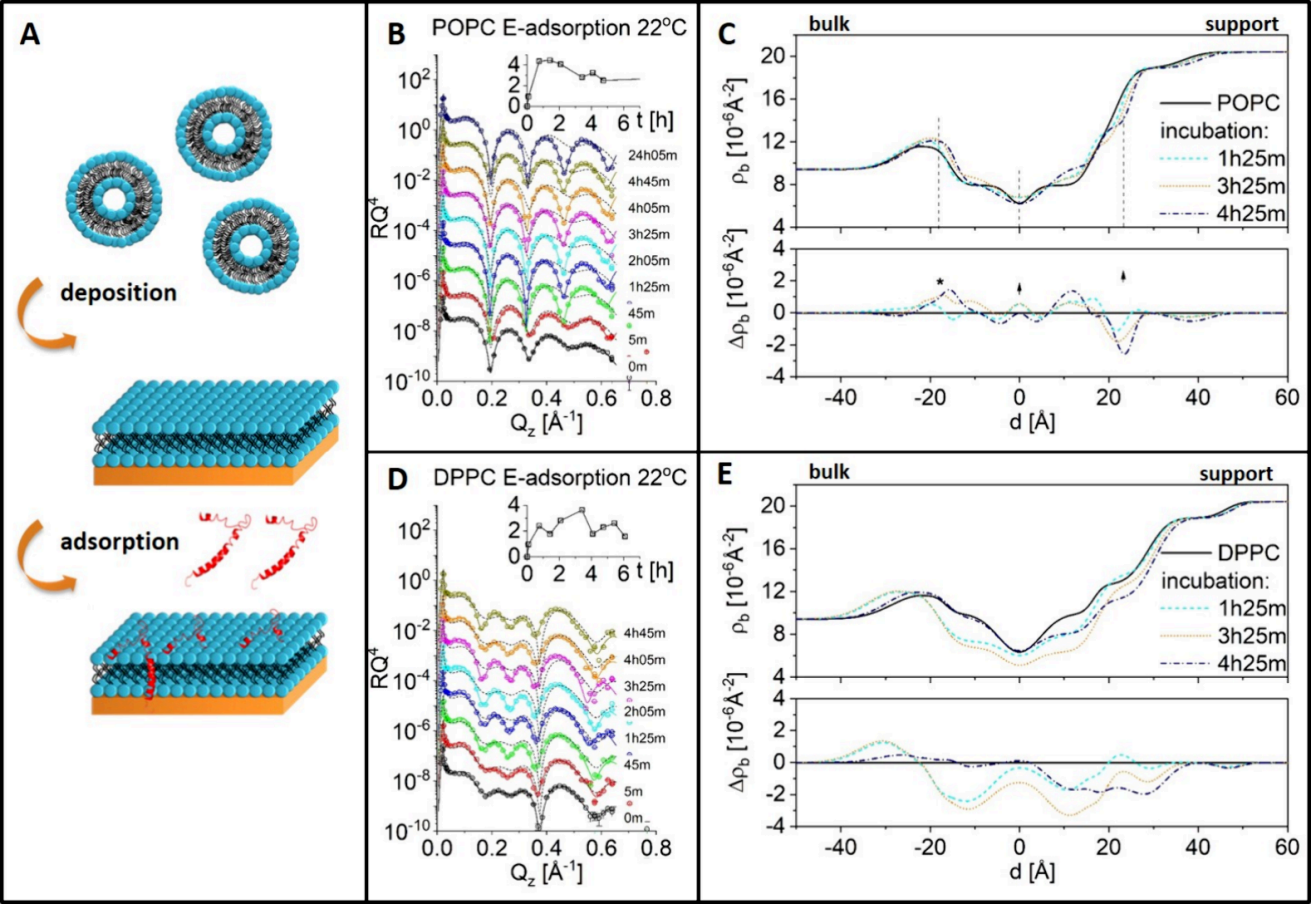
XRR data and analysis of fluid POPC (upper) and gel DPPC SLBs (lower) incubated with E protein-containing subphase. (A) Schematic illustration of the adsorption experiment. (B, D) XRR data/fit of the adsorption experiment with POPC (B) and DPPC (D), offset by a factor of 10. Dashed lines are the XRR fit of the initial state as reference. Incubation time is shown. The small inset in the XRR plot is the sum of the squared deviation of measured log(*RQ*^4^) from the initial state as a function of the incubation time. (C, E) SLD from selected incubation time points (upper) and corresponding SLD deviation Δρ_b_ from the initial pure state for the experiments with POPC (C) and DPPC (E). The complete set of SLD profiles is found in Supporting Information Section 4.

**Figure 4:**
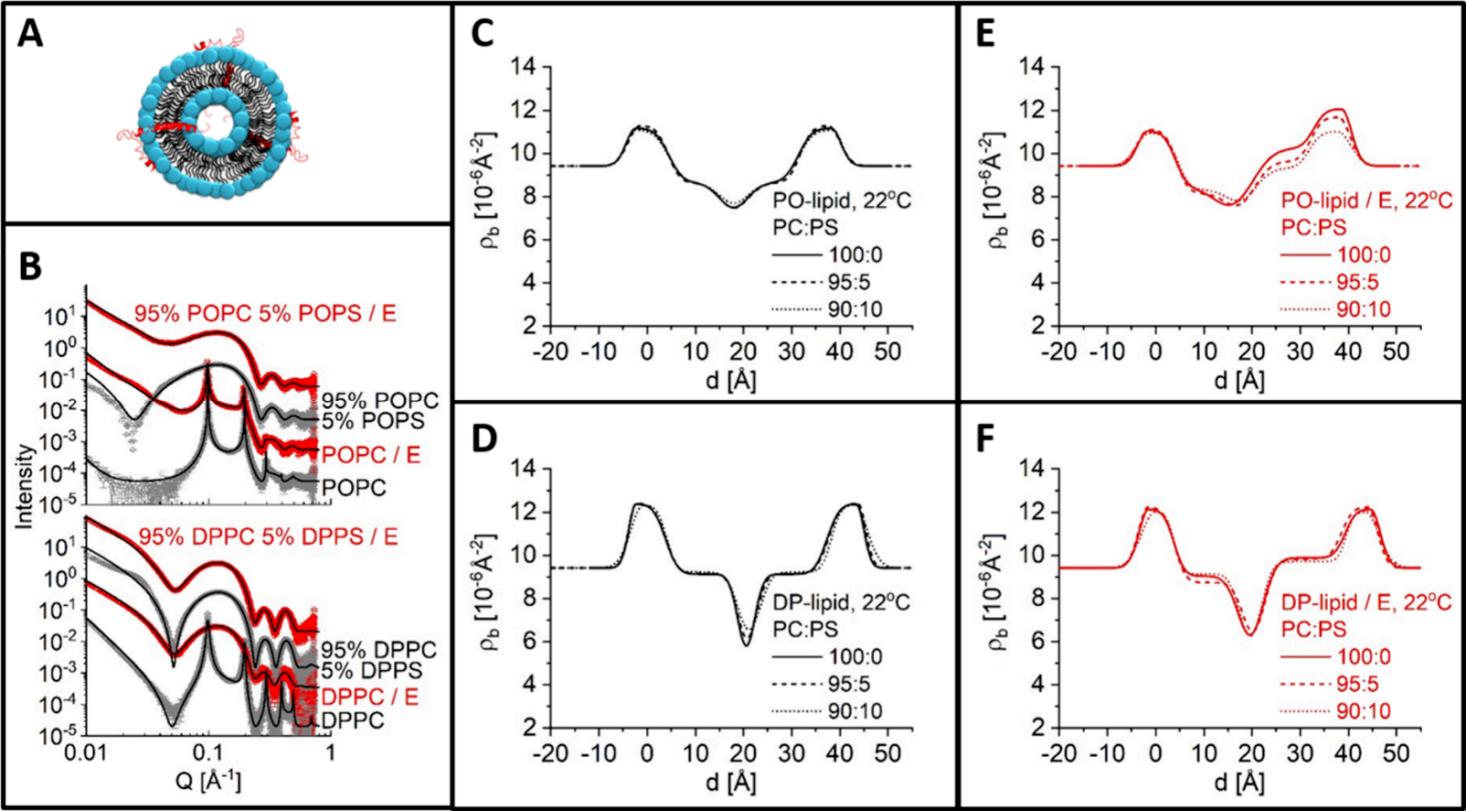
Schematic view of a liposome with incorporated E protein (A), example of the measured SAXS data from liposome dispersion at 22 °C (B), and SLD profiles obtained from the fitting (C–F). POPC and DPPC reference data are reproduced with permission from ref (28). Copyright 2023 International Union of Crystallography.

To study the structural impact of the E protein on membranes with different degrees of freedom that are physiologically relevant, we used two model membrane systems: supported lipid bilayers (SLBs) and vesicle dispersions as freestanding bilayers. SLBs on silicon (100) wafers facing aqueous buffer solutions (10 mM Tris, 0.05 mM EDTA, pH 7.4) were used to study the impact in a simplified model of a planar bilayer, where the deformation freedom along the membrane normal is limited by the solid substrate. SLBs were prepared by vesicle fusion using sonicated liposome dispersions^33,34^ and were studied by X-ray reflectometry (XRR). E protein was incorporated into SLBs in two different ways, representing two modes of interaction. The first mode consisted of co-deposited lipid bilayers with 0.5 mol % pre-incorporated E protein and reflects the natural hosting situation of E protein, which is inserted into the ERGIC lipid bilayer during translation. This was prepared by co-hydrating dry mixtures of model membrane lipids and E protein to yield liposome dispersions already hosting E protein in the lipid bilayer^2,3^ for the SLB preparation (Figure 2a). The second mode consisted of the adsorption of E protein from the aqueous bulk on peptide-free SLBs. This allowed monitoring of the incorporation of E protein by sequential XRR over a period of 5 h (Figure 3a). This scenario provides information on the membrane affinity of the E protein, although it does not represent a natural mechanism. In addition to the SLB model, vesicle dispersions in aqueous bulk under the same conditions were studied by small-angle X-ray scattering (SAXS) (Figure 4a). This model allows membranes to retain flexibility, thus mimicking their physiological state. As in SLB co-deposition experiments, membranes containing E protein were prepared by co-hydration (Figure 4a). The α-helical conformation of E protein incorporated via co-hydration in model membranes was confirmed by CD measurements after vesicle preparation (Supporting Information Section 2).

The analysis of XRR data from SLB provides the scattering length density (SLD) profile along the bilayer normal. We chose POPC bilayers to represent the physiologically relevant unsaturated lipid membranes in their fluid state (22 °C), while DPPC bilayers were used to represent saturated membranes in both gel (22 °C) and fluid phase (53 °C).^35^ (Supporting Information Section 7 compares experimental temperatures with phase transitions measured by DSC of DPPC in the presence and absence of 0.5% E protein and POPC in the presence of 0.5% E protein.) The structural change of POPC bilayers with the E protein is clearly revealed by the shift of the Kiessig fringe of the XRR data (Figure 2b, arrows). The SLD of the bilayer increased in both the head and core regions (Figure 2c and Table 1). Because the intrinsic SLD contribution from E protein is negligible at the mole fraction tested, this change is attributed solely to the modifications induced by the E protein (see Supporting Information Section 1). The area per lipid calculated from the SLD (∼52 Å^2^) was lower than the 62 Å^2^ in pure POPC bilayers. The increased SLD and the reduction of *Â* imply that a tighter packing of lipid occurs upon the insertion of E protein (Table 1). Moreover, the headgroup region of the leaflet near the support (Figure 2c, *z* ≈ 20 Å) was also modified. The SLD profile of the pure POPC SLB drops from the value of the oxide layer (18.9 × 10^–6^ Å^–2^) smoothly down to the value of the tail region (7.6 × 10^–6^ Å^–2^), where the headgroup slab between them is almost not recognizable. In the presence of the E protein, this headgroup slab becomes clearly visible around an SLD of 12.9 × 10^–6^ Å^–2^ (Figure 2c). In contrast with POPC, the structure of DPPC bilayers with E protein did not change in the gel or in the fluid phase (Figure 2d). The difference between the data sets is within the deviation among different DPPC samples (see Supporting Information Section 3). In summary, the replacement of a palmitoyl chain with an oleoyl chain enhanced the structural impact of the E protein on the SLB.

**Table 1:** Structural Parameters of SLB without and with Co-deposited E Protein

lipid	POPC		DPPC			
*T* [°C]	22		22		53	
*f*_E_ [%]	0	0.5	0	0.5	0	0.5
*d*_HH_ [Å]	41.5	42.3	47.1	48.5	43.3	42.2
*d*_core_ [Å]	31.5	31.8	33.2	33.2	31.6	31.5
ρ_b,H_ [10^–6^ Å^–2^]	11.4	12.6	11.6	12.0	12.3	11.9
ρ_b,core_ [10^–6^ Å^–2^]	7.6	9.1	8.7	8.4	8.0	7.5
Â [Å^2^]	62.2	51.5	46.6	48.3	53.8	57.7

Additionally, we tested the membrane affinity for E protein by exposing DPPC and POPC bilayers to excess bulk peptide concentrations for a prolonged time. Greater structural changes were observed in both fluid POPC and gel DPPC SLB after exposure to the protein over a time course of 5 h, as shown by changes in the XRR curve (Figure 3a, inset). The modification on the POPC bilayer is a two-step process. Within the first 45 min, its headgroup region near the wafer becomes visible (*z* ≈ 25 Å, Figure 3b), similar to the co-deposited case. This feature remained throughout the course of the measurement. The overall structure of the bilayer continuously evolves slightly over the following 4 h: the headgroup SLD of the outer leaflet becomes slightly higher than that of the lipid film in the absence of E protein; the SLD of the hydrophobic core slightly increases and becomes more homogeneous, exhibiting a reduced SLD dip at the interface of the two leaflets. However, the final SLD value after 5 h is still not as high as the SLD of the co-deposited POPC bilayer with 0.5% E protein. The structural change of the DPPC gel phase film (22 °C) as the result of E protein adsorption (Figure 3) differs from the observation under the co-deposition mode. The SLD of the whole hydrophobic core region decreased by 20% in the first 3 h. Thereafter, the two leaflets of the bilayer became asymmetric. The change observed under these experimental conditions reveals a strong affinity of the E protein for the membrane that drives its adsorption and incorporation into the lipid bilayer. The difference from the co-deposition mode is due to the excess E protein availability from the bulk over a long exposure time.

In summary, the XRR results on the planar supported bilayers show that the E protein by itself is lipophilic, consistent with its incorporation in synthetic planar bilayers in channel activity studies.^9^ Incorporation of the peptide into the membrane markedly condenses the POPC bilayer, but it is not sufficient to modify the structure of the saturated DPPC bilayer in both the gel and fluid states.

The vesicular model membrane also contained 0.5 mol % pre-incorporated E protein. In addition to the PC lipid, mixtures of PC and phosphatidylserine (PS) lipids were prepared to determine the effect of a negatively charged membrane surface on the peptide to provide a simple mimic of the ERGIC–Golgi apparatus membranes.^18,20^ It was not possible to use PC/PS mixtures in the SLB experiments due to inadequate deposition on the support caused by the negative vesicle charge. High-resolution structures of the membranes were obtained from the detailed analysis of SAXS data, using a similar method to the volumetric approach suggested by Nagle et al.,^29,36^ combined with reference data obtained from grazing incidence off-specular scattering measurements on the monolayers with the same lipid composition.^28^ The POPC membranes with up to 10% POPS (PO-membranes) were symmetric in the absence of E protein and presented similar structures (Figure 4c,d). Incorporation of the E protein resulted in asymmetry between the two leaflets. One leaflet (index 2) appears more condensed, with a smaller area per lipid and higher SLD, while the other (index 1) is expanded, with a larger area per lipid and reduced SLD (Table 2 and Figure 4e). Such an asymmetrically condensed profile (e.g., *Â*_2_ = 44.6 Å^2^, *Â*_1_ = 80.0 Å^2^ for POPC/E protein) suggests E protein curves the membrane, where one of the leaflets is squeezed due to the increased curvature while the other is being stretched. This feature decreased with increasing PS content and, consequently, surface charge density (Figure 4e), most probably because the repulsive force from the PS headgroup charge counteracted the condensation of one leaflet. No transition peak is observed between 10 and 60 °C in the DSC curve (see Supporting Information Section 7) of the POPC membrane with E protein, suggesting that the condensed lipid packing induced by the E protein does not change up to 60 °C. Note that PO-membranes alone are always fluid above 0 °C.^35^ This asymmetric condensation effect of the peptide is much weaker on the DP-bilayers. The area per lipid and the SLD were rather similar to the reference DP-layers without E protein. This is consistent with the DSC result that show no significant effect of the presence of 0.5% E protein on the phase transitions (pre and main transitions) of the DP-vesicles (see Supporting Information Section 7). The effect on the lipid packing in a condensed membrane is weak. The difference in the impact of the E protein on PO- and DP-host matrices is qualitatively consistent with the observations in SLB systems.

**Table 2:** Area per Lipid in the Membrane of the Liposomes and the Surface Charge Density, Obtained from the SAXS Analysis^a^

membrane	POPC				POPC/POPS 95/5				POPC/POPS 90/10			
*f*_E_ [%]	0^b^	0.5			0	0.5			0	0.5		
leaflet	1, 2	1	2	av	1, 2	1	2	av	1, 2	1	2	av
*Â* [Å^2^]	64.8	80.0	44.6	62.3	63.7	73.7	52.0	62.8	64.3	72.0	55.8	63.9
ρ 10^–3^[e/Å^2^]	0	0	0	0	0.78	0.68	0.96	0.80	1.56	1.39	1.79	1.56

membrane	DPPC				DPPC/DPPS 95/5				DPPC/DPPS 90/10			
*f*_E_ [%]	0^b^	0.5			0	0.5			0	0.5		
leaflet	1, 2	1	2	av	1, 2	1	2	av	1, 2	1	2	av
*Â* [Å^2^]	45.5	47.6	41.7	44.6	45.1	47.5	41.7	44.6	44.3	46.7	42.0	44.3
ρ 10^–3^[e/Å^2^]	0	0	0	0	1.12	1.14	1.15	1.14	2.25	2.36	2.36	2.36

a The looser and tighter packed leaflets of the asymmetric membranes are indexed as 1 and 2, respectively. The values averaged over two leaflets are also entered.

b Own data published elsewhere.^28^

The PS-free models also allow an interpretation at the mesoscopic scale because distinct Bragg peaks were visible, indicating multilamellar structures for the peptide-free DPPC and POPC vesicles. The membrane organization was changed in both the DPPC and POPC systems, although the membrane structures of only the POPC bilayers were modified. This is evidenced by the intensity of the Bragg peaks in the SAXS data (Figure 4b). The presence of E protein weakens the long-range order of the stacked POPC lamellae, as suggested by the smaller Bragg peaks, and completely impairs the long-range order of the DPPC lamellae because Bragg peaks were absent. None of the systems with PS showed any evidence of ordered lamellar stacking.

**Figure 5:**
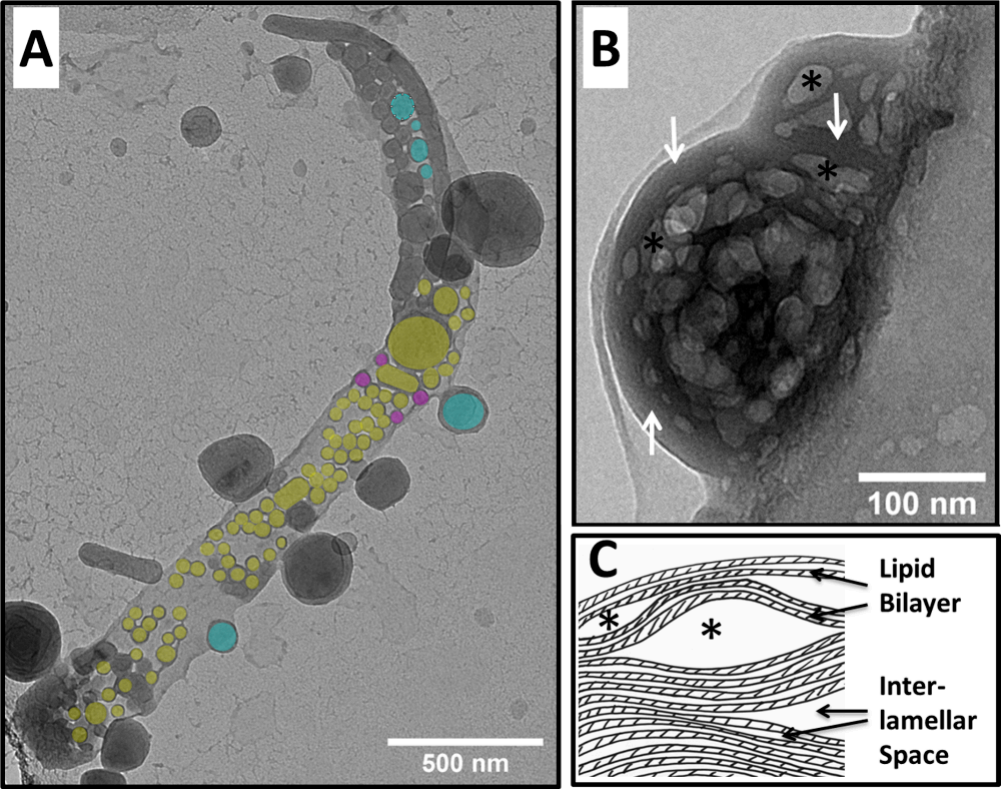
Cryo-TEM images of vesicles with the integrated E protein. (A) DPPC with E protein, intravesicular vesicles are indicated in yellow, vesicles which seem to shed outward are indicated in blue and vesicles which seem to shed into the membrane compartment in magenta. The complete image set and images without highlighted vesicles can be found in Figure S7. (B) POPC with E protein. The multilamellar system seems to be disturbed and interlayer cavities occur (examples indicated by stars). White arrows indicate multilamellar structures. (C) POPC with E protein. Model of multilamellar system with interlamellar cavities indicated by stars.

Cryo-TEM was used to directly visualize membrane organization using the SAXS samples of the model membranes in the absence of PS. DPPC samples lose their long-range lamellar stacking order upon the incorporation of E protein while for POPC samples multilamellar order is still present (Figures 5 and S5), consistent with SAXS observations. The multi- or paucilamellae of the DPPC bilayers are no longer present. Small vesicles of 50 nm diameter can be seen (yellow structures, Figure 5A), often entrapped in larger tubular lipid compartments. These tubular structures also appeared to have vesicles attached both externally (blue structures, Figure 5A) and internally (magenta structures, Figure 5A) to the tubule membrane. This morphology hints to some intermediate structure possibly related to vesicle budding-like events which have been reported to happen during SARS-CoV infections at the cellular budding site.^37^ The POPC bilayer with the E protein still preserves some multilamellar stacks that are highly irregular (Figure 5B, white arrows). The multilamellar structures are interrupted by regions with high interlamellar spacing forming cavities, although it cannot be excluded that those structures are actually entrapped vesicles (Figure 5B, stars, and Figure 5C). Both scenarios are related to the condensation and packing effect of E protein on lipid bilayers of POPC organized in a lamellar phase. This structure is very stable over time and also under electron beam irradiation, displaying little of the radiation damage typically occurring with samples of high material density. This structural stability and irregularity are best attributed to a condensed, rigid, and highly curved membrane at nanoscopic scale, which are suggested by the XRR and SAXS studies. The interlamellar cavities were absent in POPC vesicles without E protein (Figure S8).

The transmembrane E protein of coronavirus is thought to participate in virion encapsulation by altering the ERGIC membrane curvature to surround the 80 nm viral core.^38^ It has previously been reported that both the SARS-CoV-2 E protein and the homologous SARS-CoV-1 form concentration-dependent pentameric viroporins in model membranes, which can act as ion channels.^3,39,40^ To date, this has been the focus for most experimental studies^41^ while the morphology of the host membrane of the peptide has only been examined using *in silico* simulations.^16,17,42^ Herein, an experimental approach focuses on the effect of the E protein on model host intracellular lipid bilayers. Our affinity experiments demonstrate that the E protein incorporates spontaneously in lipid membranes over a long time at a high bulk concentration. The effects of E protein adsorption on the structure of lipid bilayers are marked and independent of the phase state of the lipid bilayer (POPC vs DPPC, Figure 3). However, understanding the peptide–lipid interaction requires more defined conditions. To this end, we also chose to directly incorporate the E protein into lipid bilayers during vesicle production, a scenario closer to its natural translational insertion into the ERGIC membrane. Furthermore, a defined, low peptide/lipid ratio (0.5 mol %) was used to ensure a sufficiently large lipid matrix (∼130 nm^2^) surrounding each incorporated peptide monomer (see Supporting Information Section 1), such that the results primarily reported on the structural morphology of the host lipid membrane.

The combination of the two methods suggests that SARS-CoV-2 E protein induces structural modifications at both nanometer (SAXS, XRR) and mesoscopic (Cryo-TEM, SAXS) length scales that are strongly related to the level of saturation of the acyl chains and the amount of peptide present in the membrane. Once incorporated at a low peptide/lipid ratio (0.5 mol %), fluid, unsaturated PC chains, representing the closest model for the ERGIC membrane, are subjected to an evident asymmetric condensation by 15% of the area per lipid on one leaflet (SAXS, Table 2 and Figure 4). The condensation was cross-verified by a similar observation on the supported lipid bilayer (XRR, Table 1), where the curvature freedom was eliminated. The addition of the negatively charged lipid phosphatidylserine to the unconstrained bilayers (SAXS) reduces the condensation effect on the unsaturated PO system. This can most probably be attributed to the repulsion between adjacent negatively charged PS which prevents further condensation, as even at 10 mol % PS, each PS head will have at least one other PS in the next-nearest-neighbor shell. Consequently, the asymmetry is reduced. Still, the effects are preserved to a significant extent. Conversely, the gel state of saturated lipids (DPPC) does not allow the E protein to exert its putative membrane-altering activity. This inhibitive tendency, however, is broken when the E protein was allowed to challenge the membranes at excess concentration and for longer time periods.

The result of the CD measurements shows that the α-helix fraction formed by the E protein changes with lipid matrix fluidity. It is postulated that E protein exerts its activity in its full transmembrane α-helical conformation.^43^ E protein readily adopts the active transmembrane α-helix structure in PO bilayers, irrespective of the amount of negatively charged lipids. The active conformation is seemingly suppressed in DPPC membranes, possibly due to the stiffer lipid gel phase. Proportional increments of DPPS raised the fraction of α-helix to a comparable level to that observed in the POPC/POPS membrane, suggesting that the charges might aid the E protein folding into its active form within more ordered lipid structures. However, this is not sufficient to allow detectable modifications of lipid packing at the nanoscopic scale. Nevertheless, the helical content is not the main reason for bilayer asymmetry; rather, the interplay between the type of phospholipids with the E protein in a suitable conformation seems to be necessary; otherwise, no difference of the E protein induced lipid packing alteration would have been observed for the POPC/POPS systems.

The observed effects of the E protein on lipid packing may be of biological relevance. A stable, high curvature is needed for membrane modulations, e.g., vesicle shedding or budding processes, in particular to fit the topology of the 80 nm viral core.^44^ The mostly fluid phase membranes of the ERGIC might be susceptible to E protein regardless of the PS content and in a way that favors the curvature and budding processes during viral replication. Indeed, MD simulations have shown that E protein both elicits curvature formation and localizes within curved regions in model ERGIC membranes.^17^

How pronounced the asymmetry has to be to achieve biologically relevant effects remains an interesting question. Cryo-TEM shows distinct morphological changes for DPPC and POPC. The effect on DPPC vesicles has a lot of parallels to the vesicle shedding process in biomembranes, despite the unchanged nanoscopic bilayer structure. The mesoscopic effect on the POPC membranes was even more dramatic. A highly stable structure with several interlamellar cavities was formed (Figure 5). Hence, they are consistent with the effect of the characteristics of E protein doped POPC membranes observed by SAXS and XRR, namely, a more rigid system with higher curvature resulting from an induced bilayer asymmetry. These characteristics of a stable and highly curved bilayer would be energetically beneficial for the viral budding process.

To our knowledge, the structural studies described are the first experimental studies using controllable model systems which support the role of E protein in SARS-CoV-2 in membrane modification which can result in viral budding, a theory postulated from biological experiments and MD simulations.^1,16,17,37^ Ongoing research should focus on the concentration dependency of these membrane modifications and the influence of viroporin formation through E protein oligomerization on this effect.

## Conclusion

The E protein of SARS-CoV-2 induces structural changes in model membranes that mimic intracellular biomembranes, such as those in the ERGIC. The effect is much stronger for membranes bearing unsaturated alkyl chains. The data give strong evidence that the induced structural changes can induce membrane curvature changes, which might support the role of E protein in the budding process of the virus.

## Supporting Information Available

Estimation of SLD and area contribution of the E protein to the bilayer, circular dichroism results, deviation of the supported lipid bilayers at various footprints and from different preparation, scattering length density profiles of the supported lipid bilayers during E protein adsorption, cryo-transmission electron microscopy results, structure and surface charge density of PC/PS membranes in vesicle form, differential scanning calorimetry results (PDF)
