# Supplementary material for: Membrane Condensation and Curvature Induced by SARS-CoV-2 Envelope Protein
Source: Langmuir. 2024 Jan 23;40(5):2646–55. doi: 10.1021/acs.langmuir.3c03079 (PMC10851660; doi:10.1021/acs.langmuir.3c03079)
Supplement: Supplementary file 1 — la3c03079_si_001.pdf [file la3c03079_si_001.pdf]

Supplementary Information

Membrane condensation and curvature induced by SARS-CoV-2 envelope protein

Christian Wölk[1#], Chen Shen[2#], Gerd Hause[3], Wahyu Surya[4], Jaume Torres[4], Richard D. Harvey [5]*, Gianluca Bello[5]*

1 Pharmaceutical Technology, Medical Faculty, University Leipzig, Eilenburger Straße 15a, 04317 Leipzig, Germany

2 Deutsches Elektronen-Synchrotron DESY, Notkestr. 85, 22607 Hamburg, Germany

3 Biocenter, Martin-Luther University Halle-Wittenberg, Weinbergweg 22, 06120 Halle (Saale), Germany

4 School of Biological Sciences, Nanyang Technological University, 50 Nanyang Avenue, Singapore 639798, Singapore

5 Division of Pharmaceutical Chemistry, Department of pharmaceutical sciences, University of Vienna, Josef-Holaubek-Platz 2, UZA 2, Vienna, 1090, Austria

[#]: these authors contribute equally, Chen Shen and Christian Wölk

*: corresponding authors

Gianluca Bello: gianluca.bello@univie.ac.at

Richard D. Harvey: richard.harvey@univie.ac.at

1 Estimation of SLD and area contribution of the E protein to the bilayer

Area occupied by the helix of the transmembrane domain in a lipid bilayer:

approximately 60 Å^2 for a lipid and alpha helix d~10 Å (see figure below), $\pi r^2 \approx 80$ Å^2,

with a protein content of 0.5 mol % in a bilayer → 1.3 area% are occupied by the helix

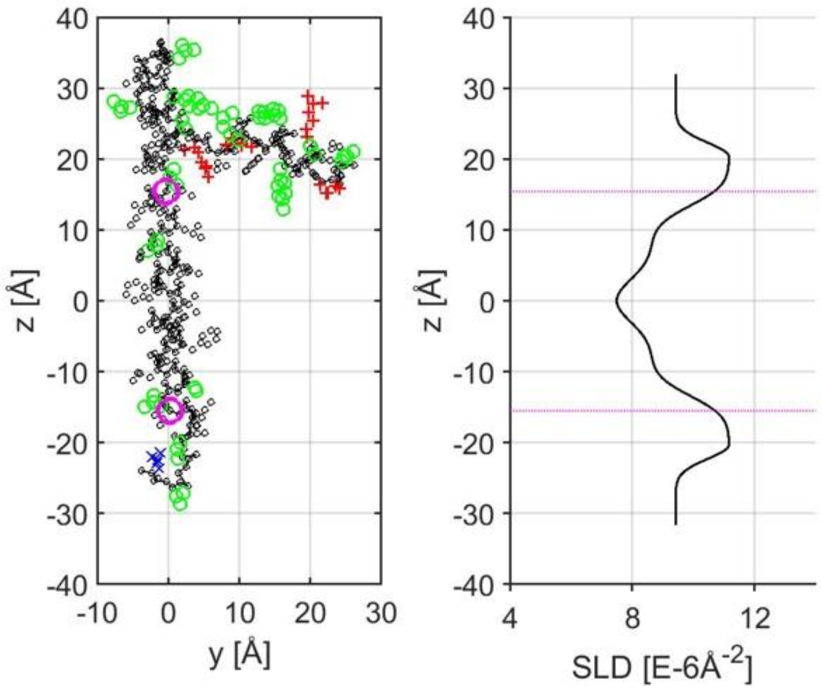

Figure S1. Side view of the E protein structure (left) and SLD profile of the bilayer of a POPC liposome (22°C) (right). The transmembrane region of E protein (residues 17 to 37, purple circles) is aligned to the hydrophobic core of the bilayer. The position of the α-carbon in these residues is indicated relative to the SLD profile (purple lines). Black circles represent the amino acid backbone and apolar residues. The red plus sign, blue cross and green circles represent polar residues with positive and negative charges, and neutral polar residues, respectively.

(a) (b)

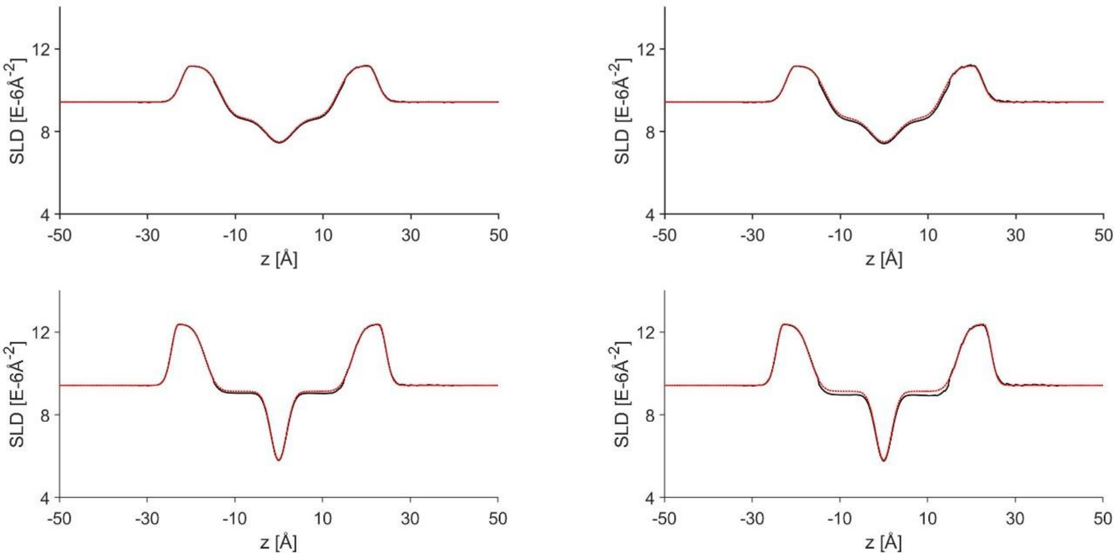

Figure S2. Calculated SLD profile of POPC bilayer (upper) and DPPC bilayer (lower) with 0.5% (a) and 1% (b) E protein, compared to SLD of pure bilayers [1] (red dashed).

Number of E protein in one 100 nm diameter vesicle:

$$N_{pep} = \frac{4\pi(500\text{Å})^2}{60\text{Å}^2} \cdot 0.5\% \approx 260$$

Membrane area around each E protein on average

$$\tilde{A} = \frac{1}{0.5\%} \cdot 65\ \text{Å}^2 + 80\ \text{Å}^2 \approx 13000\ \text{Å}^2 = 130\ \text{nm}^2$$

2 Circular Dichroism results on vesicles with incorporated E protein

The existence of α-helical structure in E protein in the model membranes was determined by circular dichroism spectroscopy. The E protein maintains a considerable percentage of alpha helix structure in all the membrane mixtures tested. Here below the content (in %) of each secondary structure of E protein calculated using the CONTIN analysis (via Dichroweb [2]). NRMSD stands for normalized root mean square deviation and represent the goodness of fitting. Ideal would be <0.05, acceptable is <0.1.

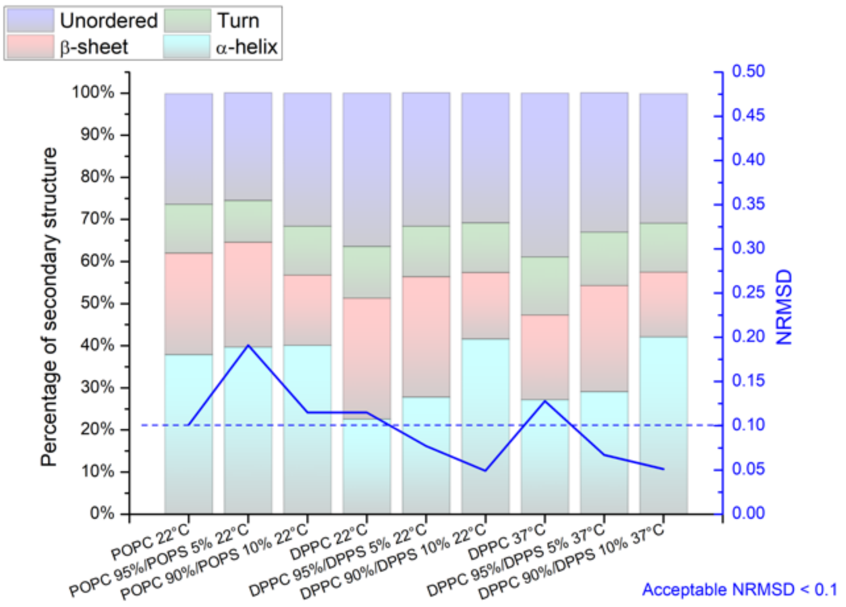

Figure S3. CONTIN analysis of the percentage of secondary structure of E protein in different bilayer systems.

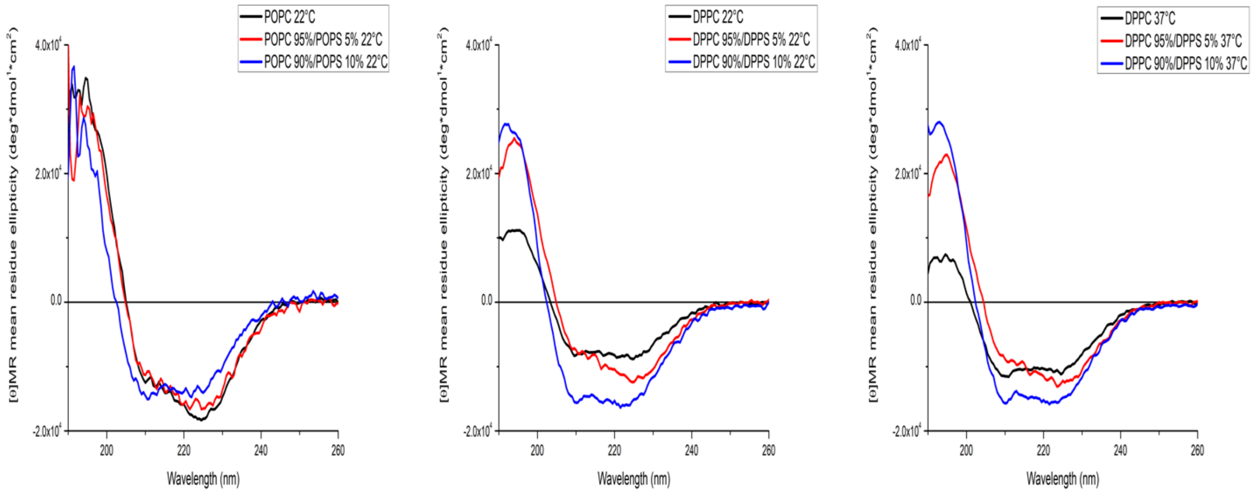

Figure S4. Circular dichroism spectra of E protein in different lipid bilayers and temperatures showing the classic α-helical pattern.

3 Deviation of the solid supported bilayers at various footprints and from different preparations

Figure S5 shows the deviation of the supported lipid bilayers at different footprints and from different preparations, in XRR data and in the analysed SLD.

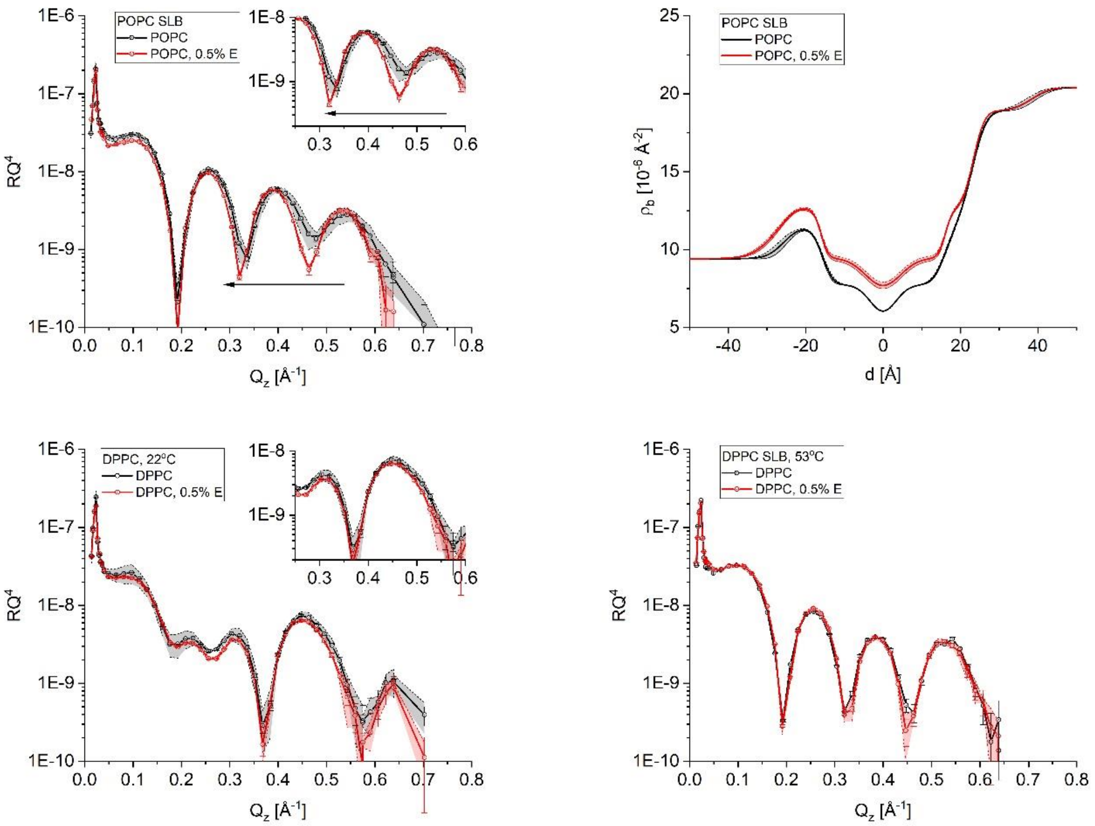

Figure S5. Deviation of the XRR of the POPC and DPPC SLBs without and with co-deposited E protein. The shaded region around the curve represent the standard deviation from multiple measurements, on different footprint and also from various preparations. Arrows show the major change on the reflectivity curve. Deviation of the SLD of the POPC-based bilayers is shown on the upper-right panel.

4 SLD profiles of SLB during E protein adsorption

Figure S6 shows the full set of the XRR data, their fit, and the obtained SLD profiles of the SLBs incubated with E protein containing aqueous phase.

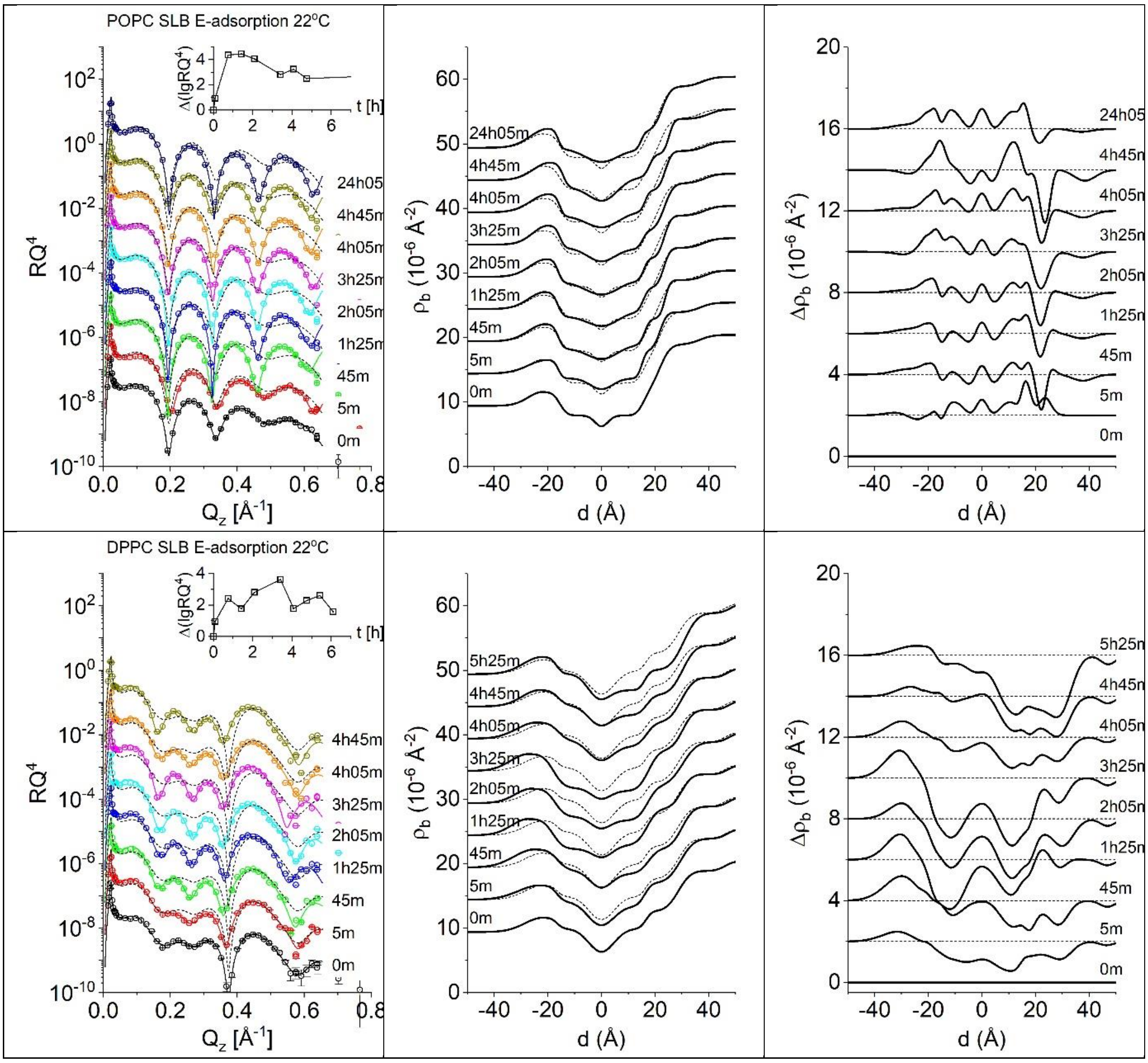

Figure S6. XRR data and their fits from the fluid POPC SLB (upper), and the gel DPPC SLB (lower), both incubated with E protein containing subphase, the obtained SLD profiles, and the SLD deviation $\Delta\rho_b$ from the initial pure state (0m). Data are offset by a factor of 10 in XRR, by 5 in SLD and by 2 in $\Delta\rho_b$. Dashed lines are the XRR fit, SLD and $\Delta\rho_b$ of the initial state. Incubation time is entered along the curves. Small insert in the XRR plot is the sum of the squared deviation of measured $\log_{10}(RQ^4)$ from the initial state, as a function of the incubation time.

5 Cryo-TEM

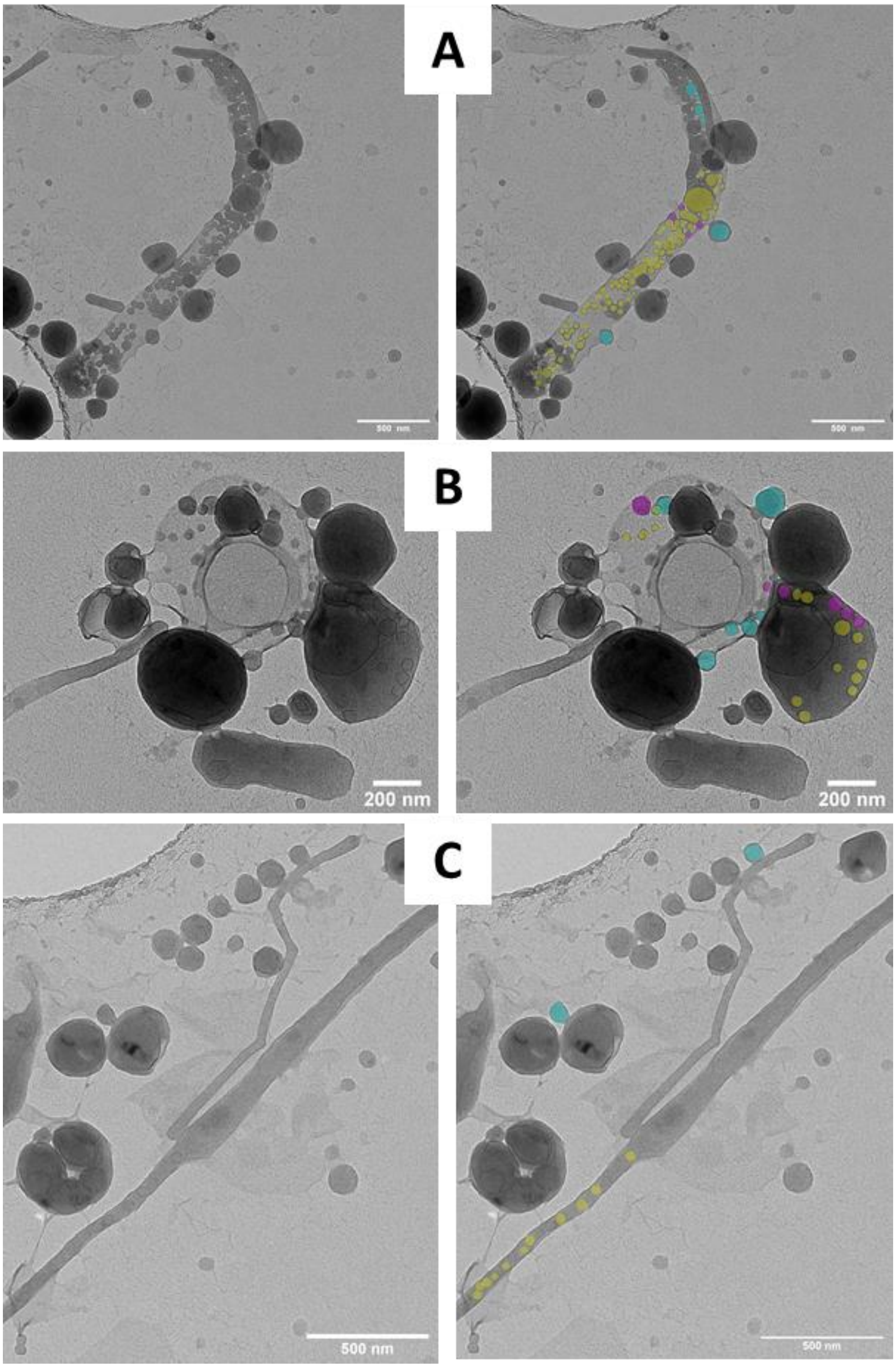

Figure S7. The complete set of cryo-TEM images of DPPC 0.5 mol% E. Intravesicular vesicles are indicated in yellow, vesicles which seem to shed outwards are indicated in blue and vesicles which seem to shed into the membrane compartment in magenta., the left image is the right without highlighted vesicles. Panel A is the same image of Figure 4A in the main text.

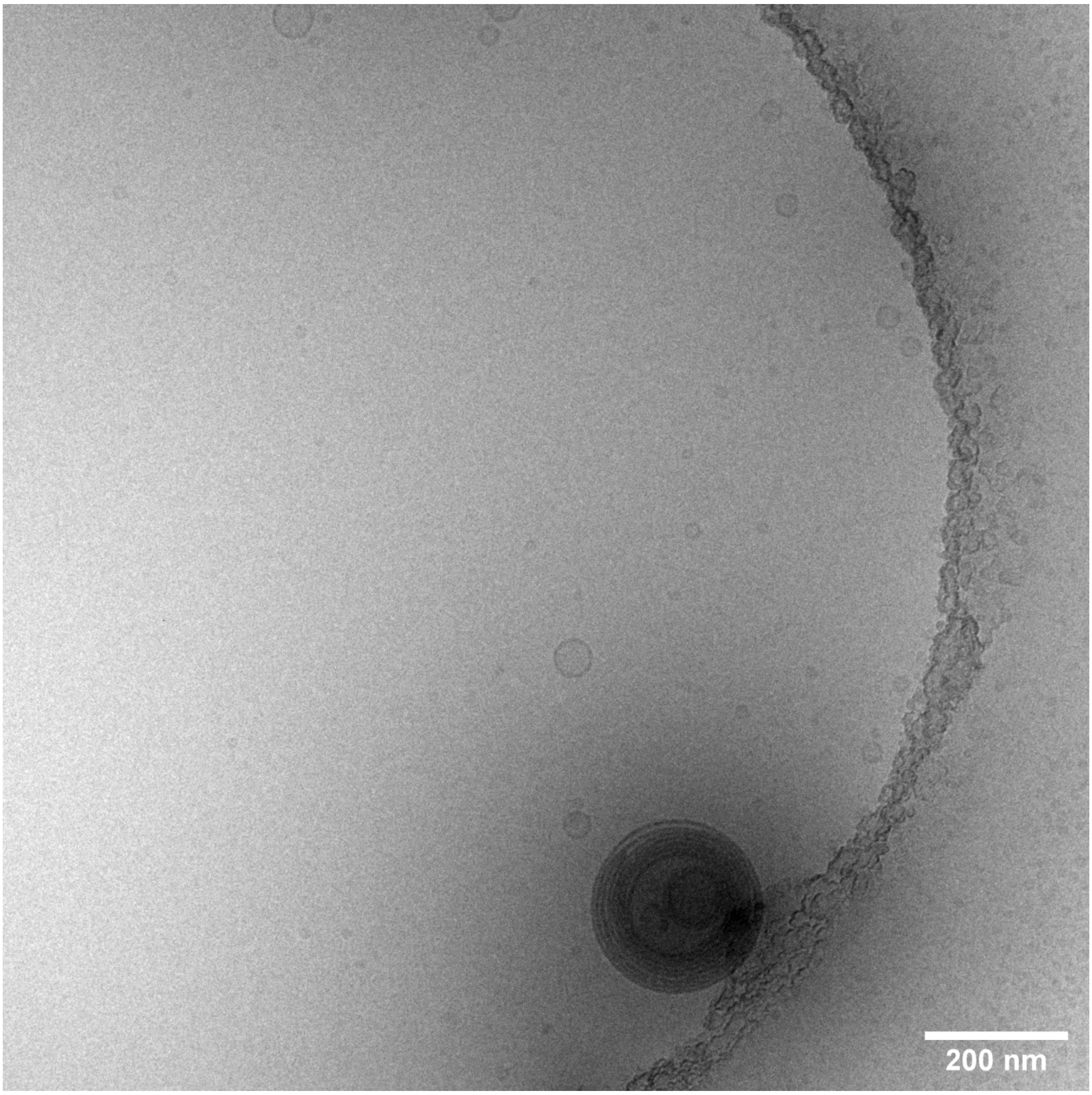

Figure S8. Cryo-TEM of POPC vesicles in absence of E protein.

6 Structure and surface charge density of PC/PS membranes in vesicle form

Table S1. Area per lipid A_L, volume V_{tail} of the hydrophobic chain region of one lipid, and the negative surface charge density obtained from the analysis of the SAXS data.
**: own data published elsewhere [1]*

membrane	POPC		POPC/POPS 95/5		POPC/POPS 90/10	
T [^{o}C]	22	37	22	37	22	37 *
A_L [Å^2]	64.8	66.1	63.7	66.2	64.3	64.2
V_{tail} [Å^3]	871.8	888.1	875.3	882.6	868.0	874.2
ρ 10^{-3}[e/Å^2]	0	0	0.78	0.76	1.56	1.56
membrane	DPPC		DPPC/DPPS 95/5		DPPC/DPPS 90/10	
T [^{o}C]	22	37	22	37	22	37 *
A_L [Å^2]	45.5	--	45.1	46.3	44.3	46.3
V_{tail} [Å^3]	763.1	--	761.8	774.6	757.6	768.3
ρ 10^{-3}[e/Å^2]	0	0	1.12	1.12	2.25	2.20

7 Differential Scanning Calorimetry of selected model systems

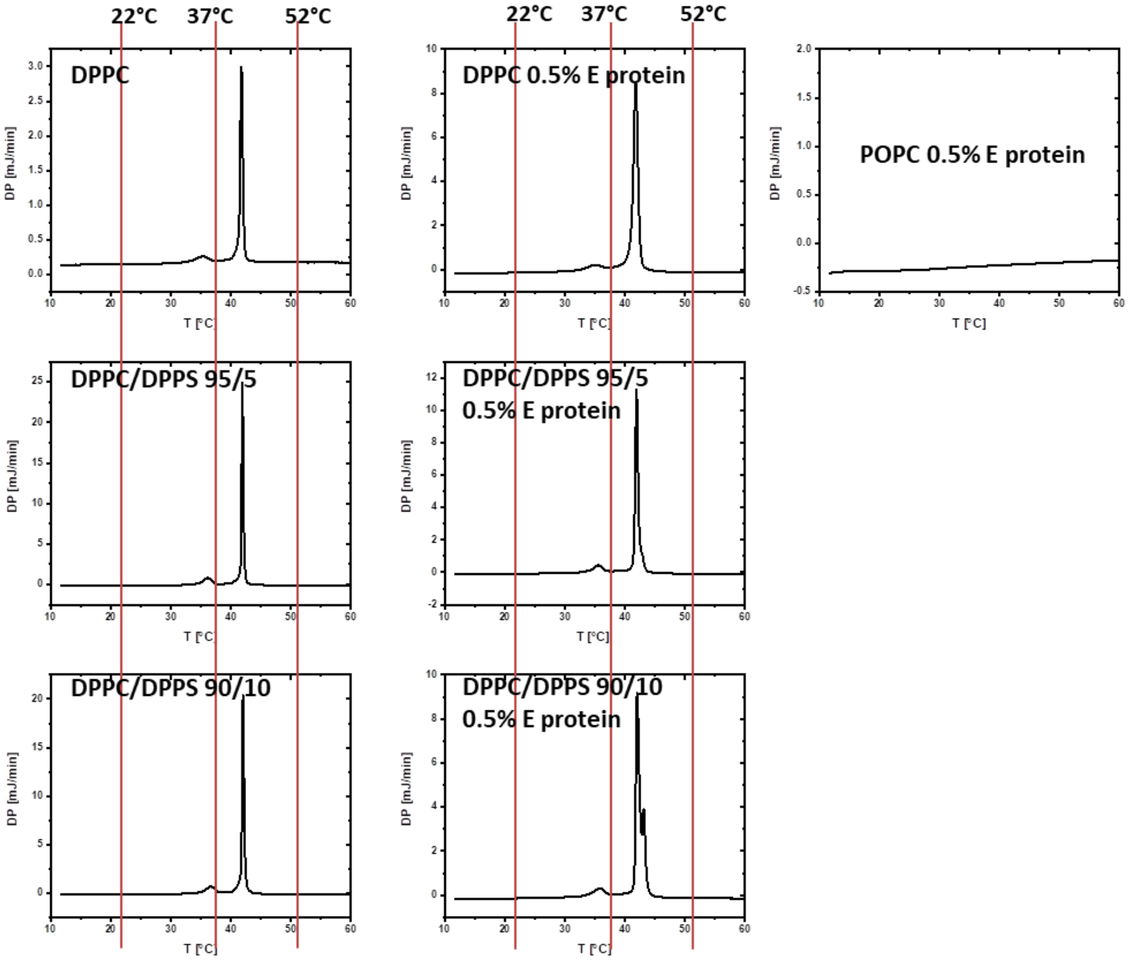

Figure S9. DSC heating curves (second scan) in the range of 10-60°C. The lines indicate the temperatures of the different x-ray experiments The temperatures indicate the temperatures of the experiments and the body temperature as orientation. No significant change of the transition temperature or peak height is observed in the DP-systems. The data from POPC in presence of E protein was performed to evaluate if the E protein in a lipid bilayer has a 1st order structural transition between the temperature of the SAXS measurement and the SLB measurement, crossing the physiological temperature. Note that POPC membrane alone does not have a 1st order phase transition above 0°C and its melt transition is below 0°C [3].

8 Structural Parameters obtained from XRR fittings

Structural parameters of the POPC bilayer and the co-deposited POPC / 0.5% E bilayer on silicon wafer, obtained from the fitting of the X-ray reflectivity data.

composition	POPC	POPC / 0.5% E
$\sigma_{6,7}$ [Å]	5.0	5.9
$\rho_{b,6}$ [10^{-6}Å^{-2}]	11.4	12.6
$\sigma_{5,6}$ [Å]	2.0	1.6
d_5 [Å]	14.8	15.0
$\rho_{b,5}$ [10^{-6}Å^{-2}]	7.8	9.5
$\sigma_{4,5}$, $\sigma_{3,4}$ [Å]	3.4	4.2
$d_{3,5}$ [Å]	1.8	1.9
d_3 [Å]	14.8	15.0
$\rho_{b,3}$ [10^{-6}Å^{-2}]	7.8	9.5
$\sigma_{2,3}$ [Å]	2.0	1.6
d_2 [Å]	6.8	7.3
$\rho_{b,2}$ [10^{-6}Å^{-2}]	11.4	12.6
$\sigma_{1,2}$ [Å]	2.9	2.4

Structural parameters of the DPPC bilayer and the co-deposited DPPC / 0.5% E bilayer on silicon wafer at 22°C and 53°C, obtained from the fitting of X-ray reflectivity data.

T [°C]	22		53	
composition	DPPC	DPPC / 0.5% E	DPPC	DPPC / 0.5% E
$\sigma_{6,7}$ [Å]	6.6	7.9	6.9	5.6
$\rho_{b,6}$ [10^{-6}Å^{-2}]	11.6	12.0	12.3	11.9
$\sigma_{5,6}$ [Å]	1.8	2.0	1.8	1.7
d_5 [Å]	14.4	14.3	14.0	13.6
$\rho_{b,5}$ [10^{-6}Å^{-2}]	10.0	9.6	8.8	8.5
$\sigma_{4,5}$, $\sigma_{3,4}$ [Å]	4.6	4.5	5.1	5.7
$d_{3,5}$ [Å]	4.3	4.5	3.5	4.4
d_3 [Å]	14.4	14.3	14.0	13.6
$\rho_{b,3}$ [10^{-6}Å^{-2}]	10.0	9.6	8.8	8.5
$\sigma_{2,3}$ [Å]	1.8	2.0	1.9	1.7
d_2 [Å]	12.1	12.3	7.6	7.9
$\rho_{b,2}$ [10^{-6}Å^{-2}]	12.7	12.5	12.3	11.9
$\sigma_{1,2}$ [Å]	3.6	3.9	2.6	2.6

Structural parameters of the POPC and the DPPC bilayers on silicon wafer, before and after 4h45min incubation with E protein from the bulk, at 22°C, obtained from the fitting of X-ray reflectivity data.

**lipid**	**POPC**		**DPPC**	
**t**ime point	**0m**in	**4h45min**	**0min**	**4h45min**
$\sigma_{6,7}$ [Å]	5.0	6.1	6.6	6.3
$\rho_{b,6}$ [10^{-6}Å^{-2}]	11.4	12.1	11.6	11.9
$\sigma_{5,6}$ [Å]	2.0	1.6	1.8	2.4
d_5 [Å]	14.8	13.4	14.4	13.6
$\rho_{b,5}$ [10^{-6}Å^{-2}]	7.8	8.3	10.0	9.6
$\sigma_{4,5}$, $\sigma_{3,4}$ [Å]	3.4	5.2	4.6	2.4
$d_{3,5}$ [Å]	1.8	4.2	4.3	2.7
d_3 [Å]	14.8	14.6	14.4	16.5
$\rho_{b,3}$ [10^{-6}Å^{-2}]	7.8	9.9	10.0	8.1
$\sigma_{2,3}$ [Å]	2.0	1.6	1.8	2.4
d_2 [Å]	6.8	7.3	12.1	13.4
$\rho_{b,2}$ [10^{-6}Å^{-2}]	11.4	13.4	12.7	11.2
$\sigma_{1,2}$ [Å]	2.9	1.6	3.6	3.8

9 References

(1) Harvey, R. D.; Bello, G.; Kikhney, A. G.; Torres, J.; Surya, W.; Wölk, C.; Shen, C. Absolute scattering length density profile of liposome bilayers obtained by SAXS combined with GIXOS - a tool to determine model biomembrane structure. *J Appl Crystallogr* **2023**, *(accepted September 2023)*. DOI: https://doi.org/10.1107/S1600576723008439.
(2) Whitmore, L.; Wallace, B. A. Protein secondary structure analyses from circular dichroism spectroscopy: Methods and reference databases. *Biopolymers* **2008**, *89* (5), 392-400. DOI: https://doi.org/10.1002/bip.20853.
(3) Curatolo, W.; Sears, B.; Neuringer, L. J. A calorimetry and deuterium nmr-study of mixed model membranes of 1-palmitoyl-2-oleylphosphatidylcholine and saturated phosphatidylcholines. *Biochimica Et Biophysica Acta* **1985**, *817* (2), 261-270, Article. DOI: 10.1016/0005-2736(85)90027-6.
